# Molecular findings and virological assessment of bladder papillomavirus infection in cattle

**DOI:** 10.1080/01652176.2024.2387072

**Published:** 2024-08-04

**Authors:** Francesca De Falco, Anna Cutarelli, Francesca Luisa Fedele, Cornel Catoi, Sante Roperto

**Affiliations:** a Dipartimento di Medicina Veterinaria e delle Produzioni Animali, Università degli Studi di Napoli Federico II, Naples, Italy; b Area Science Park, University of Salerno-Baronissi Campus, Baronissi, Italy; c Istituto Zooprofilattico Sperimentale del Mezzogiorno, Portici, Naples, Italy; d Linfa Scarl – Zona Industriale Porto Salvo, Vibo Valentia, Italy; e Faculty of Veterinary Medicine, University of Agricultural Sciences and Veterinary Medicine Cluj-Napoca, Cluj-Napoca, România

**Keywords:** Bladder tumors, bovine papillomavirus, cattle, droplet digital polymerase chain reaction (ddPCR), ovine papillomavirus

## Abstract

Bovine and ovine papillomaviruses (BPVs – OaPVs) are infectious agents that have an important role in bladder carcinogenesis of cattle. In an attempt to better understand territorial prevalence of papillomavirus genotypes and gain insights into their molecular pathway(s), a virological assessment of papillomavirus infection was performed on 52 bladder tumors in cattle using droplet digital polymerase chain reaction (ddPCR), an improved version of conventional PCR. ddPCR detected and quantified BPV DNA and mRNAs in all tumor samples, showing that these viruses play a determinant role in bovine bladder carcinogenesis. OaPV DNA and mRNA were detected and quantified in 45 bladder tumors. BPV14, BPV13, BPV2, OaPV2, OaPV1, and OaPV3 were the genotypes most closely related to bladder tumors. ddPCR quantified BPV1 and OaPV4 DNA and their transcripts less frequently. Western blot analysis revealed a significant overexpression of the phosphorylated platelet derived growth factor β receptor (PDGFβR) as well as the transcription factor E_2_F_3_, which modulate cell cycle progression in urothelial neoplasia. Furthermore, significant overexpression of calpain1, a Cys protease, was observed in bladder tumors related to BPVs alone and in BPV and OaPV coinfection. Calpain1 has been shown to play a role in producing free transcription factors of the E_2_F family, and molecular findings suggest that calpain family members work cooperatively to mutually regulate their protease activities in cattle bladder tumors. Altogether, these results showed territorial prevalence of BPV and OaPV genotypes and suggested that PDGFβR and the calpain system appeared to be molecular partners of both BPVs and OaPVs.

## 1. Introduction

Papillomaviruses (PVs) are small, non-enveloped, epitheliotropic, double-stranded DNA viruses that infect the cutaneous and mucosal epithelia in a diverse range of animals, including humans (International Agency for Research on Cancer 2007). PVs typically cause latent infections, which are characterized by the presence of viruses in the absence of clinical signs of disease (Antonsson and McMillan 2006; Maglennon et al. 2011). The establishment of a persistent PV infection is a major risk factor for cancer development and requires the virus to evade the first line of defense, the innate immune system. PVs can develop mechanisms leading to the shutdown of the host immune system, which allows them to escape immune responses, a crucial prerequisite for persistent infection, and ultimately tumorigenesis (Chiang et al. 2018; Marchetti et al. 2002). PVs slowly co-evolve with their respective hosts, showing minimal cross-transfer between species (Doorbar et al. 2015).

The bovine papillomavirus (BPV) family is composed of 44 genotypes divided into five genera (PaVE 2024). *Deltapapillomavirus* (ðPV) genus comprises four types, namely BPV1, BPV2, BPV13, and BPV14, which are highly pathogenic fibropapillomaviruses frequently associated with both cutaneous and bladder carcinogenesis of cattle (Daudt et al. 2018).

BPVs can establish persistent infections, resulting in tumors via their oncoproteins. Indeed, the BPV E5 oncoprotein has been shown to negatively regulate the host antiviral innate immune response via retinoic acid-inducible gene I-like receptors and stimulators of interferon genes in naturally occurring persistent PV infection in cattle (De Falco, Cutarelli, Gentile, et al. 2021; De Falco et al. 2022). The major oncogene product of ðPV is the transmembrane E5 protein (Talbert-Slagle and DiMaio 2009), which is the most highly conserved oncoprotein among fibropapillomaviruses (Venuti et al. 2011; DiMaio and Petti 2013; Van Doorslaer 2013). The main transforming pathway by bovine ðPV is based on E5 oncoprotein, which interacts with platelet derived growth factor β receptor (PDGFβR). Experimental studies showed that the BPV E5 oncoprotein specifically binds to the transmembrane domain of the PDGFβR (Talbert-Slagle and DiMaio 2009) and induces receptor dimerization and activation resulting in receptor autophosphorylation and the initiation of a mitogenic signaling cascade (Demoulin and Essaghir 2014; Karabadzhak et al. 2017). This protein-protein interaction occurs in spontaneous urinary bladder tumors in cattle and water buffaloes (Borzacchiello et al. 2006; Roperto et al. 2013; Russo et al. 2016). Additional minor alternative transformations of molecular pathways aided by BPV E5 have also been described. The BPV E5 oncoprotein activates calpain3 protease, which promotes the displacement of the E_2_F_3_ transcription factor from the retinoblastoma protein (pRb). E_2_F_3_ promotes cell cycle progression by modifying the cellular proliferation rate of the neoplastic bladder urothelium (Olsson et al. 2007; Roperto, De Tullio, et al. 2010). The BPV E5 oncoprotein is also responsible for altered proteostasis and severe abnormal protein degradation in bovine neoplastic urothelial cells because it binds to subunit D of the V_1_-ATPase proton pump (Roperto et al. 2014). Inactivation of V-ATPase is considered as an additional cellular transformation pathway facilitated by BPV E5 (Suprynowicz et al. 2006).

Ovine papillomaviruses (OaPVs) are involved in bladder carcinogenesis in cattle. Transcriptionally active ovine ðPVs, namely OaPV1, OaPV2, OaPV3 (an epitheliotropic dyokapapillomavirus), and OaPV4, are associated with BPV-negative bladder tumors in cattle. Therefore, OaPVs may play a role in the etiopathogenetic mechanisms of bovine bladder carcinogenesis (De Falco et al. 2023). Moreover, OaPV DNA has been detected and quantified in equine sarcoids, which suggests that these viruses may lead to a novel cross-species transmission in an additional species (De Falco et al. 2024)

Digital polymerase chain reaction is a new-generation traditional quantitative PCR (Kojabad et al. 2021). Droplet-based digital PCR (ddPCR) is currently the most accurate and sensitive method for measuring the nucleic acid load (Biron et al. 2016). Therefore, ddPCR provides more precise and reproducible detection of low-abundance pathogens in the clinical diagnosis of infectious diseases, including viral diseases (Li et al. 2018). ddPCR is believed to be the most sensitive method for diagnosing oncogenic human papillomavirus (HPV) (Isaac et al. 2017; Lillsunde Larsson and Helenius 2017) as well as BPVs and OaPVs (De Falco, Corrado, et al. 2021; De Falco, Cutarelli, D’Alessio, et al. 2021)

Although the prevalence and distribution of some BPV genotypes in the urinary bladder of cattle have been studied (Carvalho et al. 2006; Wosiacki et al. 2006; Roperto, Borzacchiello, et al. 2010a; Roperto, Munday, et al. 2016; Roperto, Russo, et al. 2016), in-depth analyses of persistent viral infection resulting in bladder tumors regarding the diversity, abundance, and characteristics of BPV, and mostly of OaPV genotypes, are still scarce.

Therefore, this study aimed to report the molecular findings and virological evaluation of BPVs and OaPVs in bladder tumors in adult cattle and investigate territorial prevalence of BPV and OaPV genotypes using ddPCR technology in an attempt to gain insights into their pathogenetic molecular pathways.

## 2. Materials and methods

### 2.1. Sample collection and processing

Fifty-two bladder tumors were collected from slaughterhouses with permission from the medical authorities. Additional twelve bladder samples were collected from healthy cattle. Portions of the samples were cryopreserved in liquid nitrogen for molecular analysis, including diagnostic procedures using ddPCR, while other portions were fixed in 10% neutral buffered formalin and processed for paraffin embedding for microscopic investigation, as previously reported (De Falco et al. 2023).

### 2.2. DNA extraction

Total DNA was extracted from each sample using a DNeasy Blood and Tissue Kit (Qiagen, Wilmington, DE, USA), according to the manufacturer’s instructions.

### 2.3. Droplet digital polymerase chain reaction (ddPCR)

For ddPCR, a QX100 ddPCR System (Bio-Rad Laboratories, Hercules, CA, USA) was used according to the manufacturer’s instructions. The primer and probe sequences for BPV1, BPV2, BPV13, BPV14, and OaPV1, OaPV2, OaPV3, and OaPV4 as well as the tool used to generate droplets and thermal profiles have been described previously (De Falco,Corrado, et al. 2021; De Falco et al. 2023). Each sample was analyzed in triplicate.

### 2.4. RNA extraction and one-step reverse transcription (RT)-ddPCR

RNA was extracted from the above samples using the RNeasy Plus Mini Kit (Qiagen, NW, DE, USA) according to the manufacturer’s instructions. This kit contains genomic DNA eliminator spin columns. RNA (100 ng) was used with the One-Step RT-ddPCR Advanced Kit for Probes (Bio-Rad Laboratories, Hercules, CA, USA) according to the manufacturer’s instructions, as previously described (De Falco et al. 2023).

### 2.5. Antibodies

Mouse antibody against calpain-1 was purchased from Sigma-Aldrich (Merck, DA, Germany). Rabbit antibodies against calpain-2 were purchased from Abcam (CA, UK). Rabbit polyclonal anti-PDGFβR and antiphosphorylated PDGFβR antibodies, as well as mouse antibodies against E_2_F_3_ and GAPDH, were purchased from Santa Cruz Biotechnology (TX, USA).

### 2.6. Western blot (WB) and densitometric analysis

WB analysis was performed on four healthy samples, four samples positive for BPV alone, and ten samples positive for both BPVs and OaPVs as reported previously (De Falco et al. 2023).

Western blot bands were analyzed and the average intensity of the bands was determined using software Image Lab Software (Bio-Rad Laboratories, Hercules, CA, USA). Data were collected in terms of average intensity of bands of, PDGFβR, pPDGFβR CALP1 and CALP2, E2F3 per average intensity of bands of GAPDH and imported to a spreadsheet (Excel; Microsoft, Redmond, WA, USA).

### 2.7. Statistical analysis

The results were analyzed to assess the significance of BPV and OaPV prevalence with Statistical Package for Social Sciences (SPSS) version 20 statistical software (IBM, Armonk, NY, USA). Categorical variables were reported as frequencies and percentages and were compared using Chi-squared and Friedman tests. Statistical significance was set at *p* ≤ 0.05.

## 3. Results

### 3.1. Virological assessment

BPV1 infection was observed in 21 of the 52 urinary bladders, accounting for ∼40.4% of the examined samples. BPV1 alone was responsible for only two infections, whereas in the remaining cases, it was involved in multiple infections. BPV1 was identified by detecting and quantifying its DNA and mRNA. Quantification of BPV1 DNA ranged from 0.1 to 8.8 copies/μL, whereas its mRNA, observed in nine samples, from 0.1 to 7 copies/μL. BPV2 DNA-containing positive samples were detected in 31 of 52 urinary bladders (∼59.6%), whereas viral transcripts were quantified in 12 of the positive samples (∼38.7%). Range of BPV2 DNA and mRNA was from 0.15 to 6.4 and from 0.09 to 5 copies/μL, respectively. BPV2 caused a single infection in five of the samples. Thirty-four bladder tumors contained BPV13 DNA (∼65.4%), and transcripts were quantified in 13 of them (∼38.2%). BPV13 DNA was detected as a single infection in five samples. Quantification of BPV13 DNA ranged from 0.1 to 45 copies/μL, whereas BPV13 mRNA ranged from 0.1 to 3.3 copies/μL. BPV14 DNA was detected in 35 of 52 bladder tumors (∼67.3%). Among these, 13 tumors contained mRNA (∼37.1%). BPV14 alone was observed in five infections. BPV14 DNA and mRNA copies ranged from 0.09 to 12.3/μL and 0.08 to 9.4/μL, respectively.

**Figure 1.**
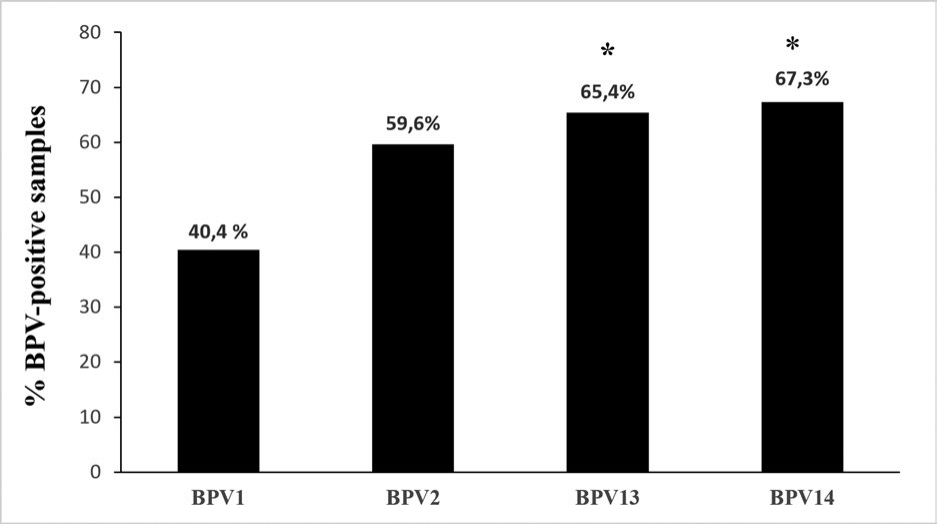
Percentages of single BPV DNA detection found in the 52 bladder tumors using ddPCR. The prevalence of BPV13 and BPV14 appeared to be significant (*) in this series of bladder tumors in comparison with BPV1 being *p* ≤ 0.05.

Figure 1 summarizes the abovementioned results, and analysis of the data showed that out of the total BPV-positive cases, the prevalence of BPV13 and BP14 was significant (*p* ≤ 0.05) in comparison with BPV1 prevalence. Supplemental Table 1 reports the raw data from all 52 samples.

**Figure 2.**
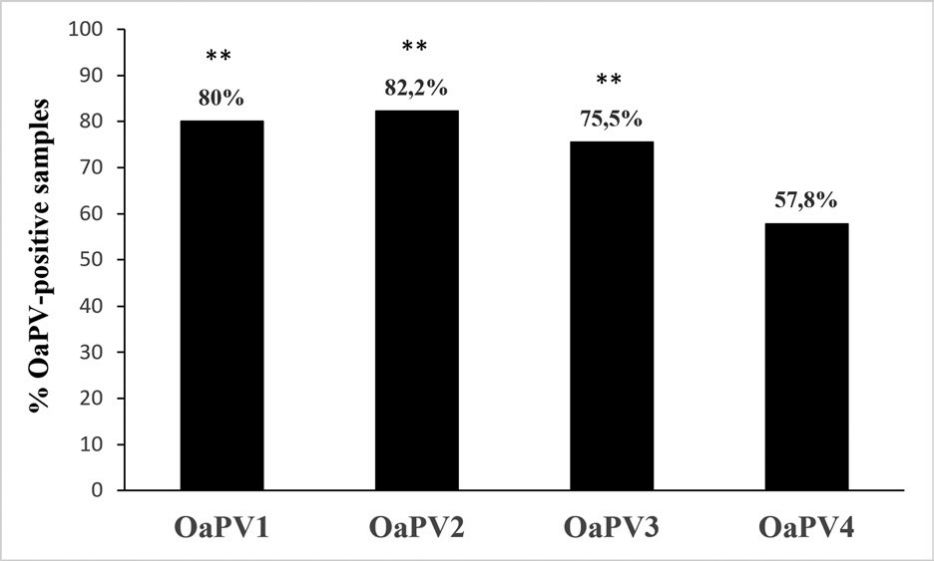
Percentages of single OaPV DNA detection found in the 45 positive samples. Significant prevalence of OaPV1, OaPV2, and OaPV3(***) in this series of bladder tumors compared to OaPV4 being *p* ≤ 0.001.

Forty-five bladder tumors harbored OaPV infection. In particular, 36 samples (∼80%) contained OaPV1 DNA, 29 of them (∼80.5%) were transcriptionally active. OaPV1 DNA ranged from 0.4 to 40 copies/μL. OaPV1 mRNA ranged from 0.1 to 6.5 copies/μL. OaPV2 DNA was quantified in 37 bladder tumors (∼82.2%) and ranged from 0.2 to 103 copies/μL. OaPV2 transcripts, ranging from 0.1 to 5.5 copies/μL, were detected in 27 samples (∼73%). OaPV3 DNA was quantified in 34 of 45 bladder tumors (∼75.5%), 23 of which were transcribed (∼67.6%). OaPV3 DNA and mRNA copy number ranged from 0.3 to 145/μL, and 0.4 to 67.7/μL, respectively. OaPV4 DNA was found in 26 tumors (∼57.8%) and transcribed in 11 of them (∼42.3%). OaPV4 DNA and mRNA copy number ranged from 0.2 to 3.5/μL and 0.1 to 2/μL, respectively. Figure 2 summarizes the abovementioned results, and the prevalence of OaPV1, OaPV2 and OaPV3 was highly significant (*p* ≤ 0.001) compared to OaPV4 prevalence. Supplemental Table 1 reports the raw data from all 45 bladder tumors where OaPV infection was identified.

### 3.2. WB analysis

**Figure 3.**
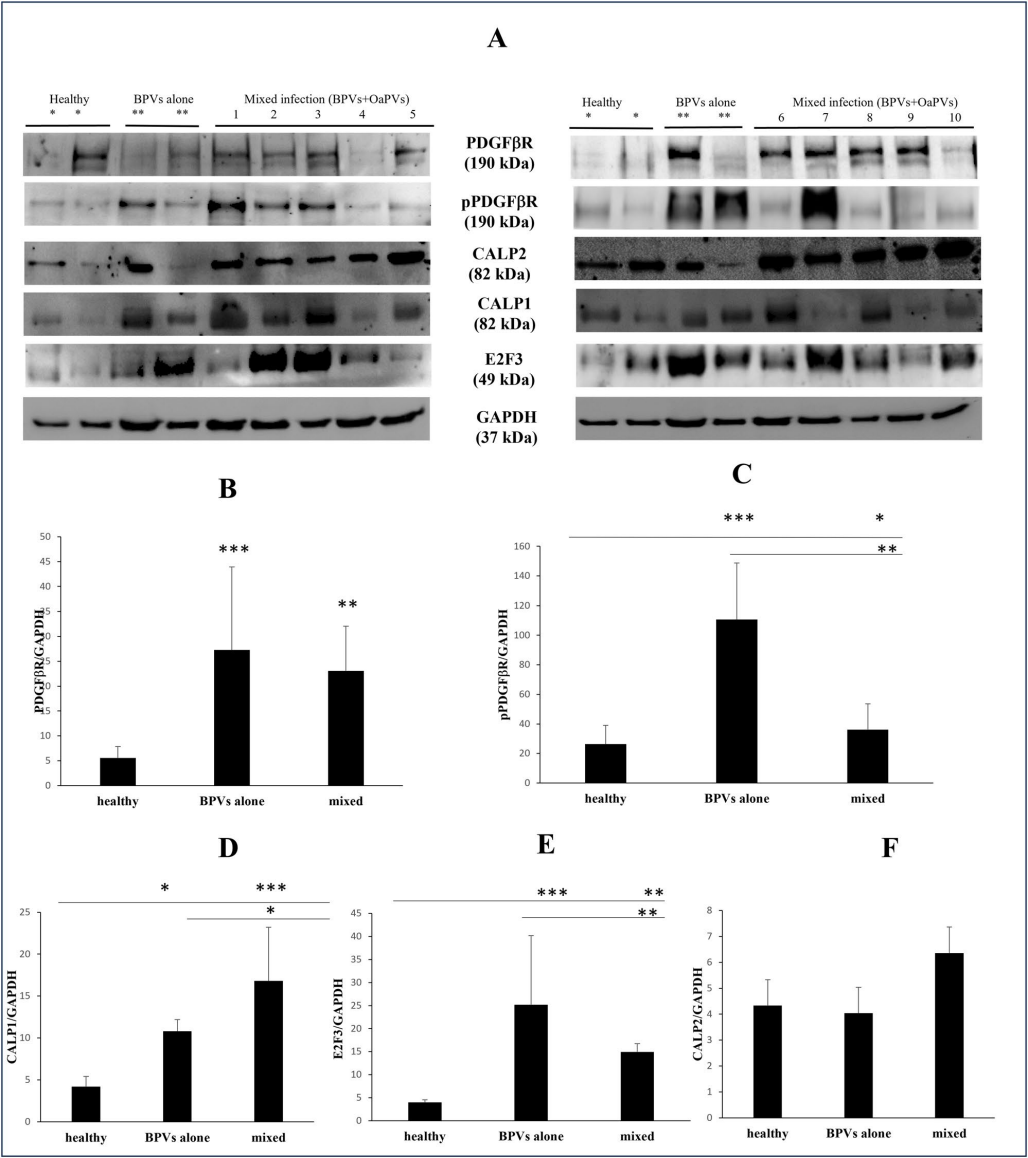
(A) WB analysis performed on healthy and pathological bladders of total and phosphorylated PDGFβR, E_2_F_3,_ calpain 1 and calpain 2. *Expression of proteins in healthy bladders as control; **expression in bladders infected by BPVs only; lines signed by 1–10: expression in bladders tumors infected both by BPVs and OaPVs. (B–C) Significant overexpression of total and phosphorylated PDGFβR in infected bladders as shown by densitometric analysis. (D) Significant overexpression of calpain 1 in infected bladders as shown by densitometric analysis. (E) Significant overexpression of E_2_F_3_ in infected bladders as shown by densitometric analysis. (F) Densitometric analysis showed no significant variation of calpain 2 expression in pathological bladders compared to healthy bladders.

Immunoblotting analysis detected a significant overexpression of total and phosphorylated PDGFβR in bladder tumors infected by BPVs alone as well as in those where a mixed infection (BPVs and OaPVs) was identified (Figure 3(A–D)). Furthermore, WB revealed significant calpain1 and transcription factor E_2_F_3_ overexpression in the above groups of tumors compared to healthy urinary bladders (Figure 3E and 3F). No significant changes were noted in calpain2 expression in bladder tumors compared to healthy bladders.

## 4. Discussion

Our study represents the first molecular assessment of BPV and OaPV infection in cattle bladder tumors using ddPCR, an improved version of conventional PCR. BPVs were found to quantify the DNA and mRNA levels of the E5 oncogene. OaPV1, −2, and −3 have been detected in many bladder tumors, quantifying non-transcribed and transcribed E5 and E6 oncogenes. OaPV2 was detected and quantified using the DNA of the L1 gene, which encodes the major structural protein of PVs, as well as its mRNA expression. This study takes advantage of the deep analysis of viral DNA and its transcript detection and quantification to expand the information about the distribution of BPV and OaPV genotypes among histologically characterized bladder samples and to explore the proportion of each genotype in mixed infections. Our study showed that bovine ðPV infection was present in all bladder tumors, which suggests that these viruses are highly pathogenic and have a role in the etiopathogenesis of bladder tumors of cattle, similar to the role of high-risk HPV in cervical cancer in women. Epidemiologically, the virological findings of this study indicated that BPV14 was the most prevalent genotype, with a prevalence as high as 67.3%. BPV14 mRNA was detected and quantified in 37.1% of infections, showing for the first time that BPV14 is transcriptionally active in urinary bladder tumors, thus representing an additional marker of active BPV infection. Although BPV14 DNA has been sequenced in neoplastic urinary bladder of cattle (Roperto, Munday, et al. 2016), as of now, BPV14 mRNA has not been detected in bovine bladder tumors (de Alcântara et al. 2021). Therefore, our findings are consistent with the role of BPV14 in the etiopathogenesis of bladder tumors in cattle and its transforming potential. BPV2 and BPV13 are frequently detected in multiple BPV infections. Our study showed the territorial prevalence of BPV genotypes and confirmed previous investigations (Roperto, Russo, et al. 2016) showing that BPV13 is as important as BPV2 in bladder carcinogenesis in cattle, at least in Italy. The association of BPV13 infection with bladder tumors in cattle is still scarce (de Alcântara et al. 2021). Only recently have the first instances of isolated BPV13 and BPV14 infections in cattle been reported in large geographical areas such as Brazil and China (de Alcântara et al. 2021; Meng et al. 2021).

Furthermore, this study showed that OaPV infection can frequently be identified in bladder tumors in cattle, which corroborates the findings of our recent study showing the potential etiological role of OaPVs in bovine bladder neoplasia (De Falco et al. 2023). Viral outcomes suggest that OaPVs are responsible for both abortive and productive infections of the bovine urinary bladder, thus showing pathogenic mechanisms superimposable to those of BPVs (Roperto et al. 2012). In the current study, OaPV1, OaPV2, and OaPV3 were the genotypes most frequently associated with carcinogenesis in the urinary bladder of cattle. Since this is the first study to report BPV- and OaPV-associated infections in bladder tumors, it is difficult to extrapolate the biological significance of this PV mixed infection. Therefore, the role of these oncoviruses in the crosstalk between molecular mechanisms leading to bladder carcinogenesis in cattle remains to be elucidated. In comparative medicine, the current evidence regarding the effect of multiple PV infections is conflicting. While some studies have shown that multiple PV infections contribute to an increased risk of cancer, others have shown that multiple PV infections do not confer additional carcinogenic effects compared to single PV infections (Adcock et al. 2019; Chen et al. 2022; Kim et al. 2022). We cannot exclude the possibility that the BPVs and OaPVs act cooperatively. Future research to better understand some molecular pathways of BPVs and OaPVs, alone and/or synergistically, is desirable. In particular, epidemiological studies are warranted to corroborate our results.

The molecular findings of this study showed that activated calpain1, an intracellular cysteine protease, was overexpressed in bladder tumors infected both by BPVs alone and/or by BPVs and OaPVs, very likely regardless the virus genotypes. It is worth noting that both bovine and ovine ðPVs display their activity through the E5 oncoprotein, known to be the most highly conserved protein of fibropapillomaviruses. It is worth noting that both bovine and ovine Activation of the calpain system may play an important role in the molecular mechanisms underlying bladder tumors caused by BPVs and OaPVs in cattle. Calpain1 is activated by HPV and OaPV oncoproteins (Darnell et al. 2007; De Falco et al. 2023). Proteomic studies showed that calpain3 is proteolytically active in BPV-associated bladder tumors (Roperto, De Tullio, et al. 2010). Calpain1 and 3 activation by PVs induces degradation of pRb, which releases E_2_F transcription factors to promote cell cycle progression in urothelial neoplasia (Darnell et al. 2007; Roperto, De Tullio, et al. 2010; Russo et al. 2016). We suggest that an activated inter-calpain network could occur in the bladder tumors in cattle, exerting their protease activity and resulting in E_2_F_3_ overexpression. Our assumption appears to be supported by recent experimental data. It has been demonstrated that calpain family members work cooperatively to mutually regulate their protease activities, with calpain3 shown to trigger a calpain cascade, thereby facilitating calpain1 activity (Ojima et al. 2023).

The high prevalence of multiple and mixed infections in our study could also reflect immunological mechanisms because all cattle in our series grazed on lands rich in bracken fern, a plant containing toxic principles such as ptaquiloside. a norsesquiterpenoid glucoside, known to be responsible for severe impairment of the bovine immune system (Roperto et al. 2010; Medeiros-Fonseca et al. 2021). Immunological mechanisms have also been hypothesized for multiple HPV infections (Chaturvedi et al. 2011). Further studies are required to characterize the immunological determinants, if any, of PV infections.

## Supplementary Material

Supplemental Material

## Data Availability

The data that support the findings of this study are available from the corresponding author upon request
