# Supplementary material for: Molecular findings and virological assessment of bladder papillomavirus infection in cattle
Source: Vet Q. 2024 Aug 4;44(1):1–7. doi: 10.1080/01652176.2024.2387072 (PMC11299453; doi:10.1080/01652176.2024.2387072)
Supplement: Supplemental Material [file TVEQ_A_2387072_SM1098.docx]

|  |  | **BPV1 E5** | | **BPV2 E5** | | **BPV13 E5** | | **BPV14 E5** | | **OaPV1 E5** | | **OaPV2 L1** | | **OaPV3 E6** | | **OaPV4 E5** | |
| --- | --- | --- | --- | --- | --- | --- | --- | --- | --- | --- | --- | --- | --- | --- | --- | --- | --- |
| **N°** | **Microscopic patterns of bladder tumors** | **DNA** | **mRNA** | **DNA** | **mRNA** | **DNA** | **mRNA** | **DNA** | **mRNA** | **DNA** | **mRNA** | **DNA** | **mRNA** | **DNA** | **mRNA** | **DNA** | **mRNA** |
| **1#** | **Non-invasive papillary urothelial carcinoma (UC), low grade** | **0.3** | **N** | **0.3** | **0.1** | **6** | **N** | **1.3** | **N** | **40** | **0.5** | **103** | **1** | **145** | **1.7** | **1** | **N** |
| 2 | Non-invasive papillary UC, high grade | 0.4 | 0.4 | N | N | N | N | N | N | 0.2 | 6.5 | N | N | 0.8 | N | N | N |
| 3 | Non-invasive papillary UC, high grade | N | N | 0.2 | N | N | N | N | N | N | N | N | N | N | N | N | N |
| 4 | Non-invasive papillary UC, low grade | N | N | N | N | 0.1 | 0.5 | 0.1 | 1 | 0.3 | 0.1 | N | N | 1 | 0.4 | N | N |
| 5 | Non-invasive papillary UC, high grade | N | N | N | N | N | N | 0.6 | N | 1.5 | N | 2.5 | N | 1 | N | N | N |
| 6 | Non-invasive papillary UC, high grade | N | N | N | N | N | N | 5.3 | N | N | N | 0.2 | 2.3 | N | N | N | N |
| **7#** | **Squamous cell carcinoma** | **1** | **0.2** | **0.5** | **0.1** | **N** | **N** | **1.3** | **N** | **1** | **0.9** | **2** | **N** | **1** | **N** | **N** | **N** |
| **8#** | **Non-invasive papillary UC, high grade** | **2.8** | **N** | **1.3** | **N** | **2.3** | **N** | **N** | **N** | **2.3** | **1.2** | **7** | **2** | **10** | **5.6** | **3.5** | **0.2** |
| **9*** | **Non-invasive papillary UC, high grade** | **N** | **N** | **N** | **N** | **0.6** | **0.5** | **2.7** | **N** | **N** | **N** | **N** | **N** | **N** | **N** | **N** | **N** |
| **10*** | **Non-invasive papillary UC, low grade** | **N** | **N** | **N** | **N** | **N** | **N** | **0.8** | **0.1** | **N** | **N** | **N** | **N** | **N** | **N** | **N** | **N** |
| **11*** | **Cavernous hemangioma** | **N** | **N** | **0.2** | **N** | **N** | **N** | **N** | **N** | **N** | **N** | **N** | **N** | **N** | **N** | **N** | **N** |
| 12 | Invasive UC, high grade | N | N | N | N | 1.8 | 1 | N | N | 2 | 2.4 | 1 | 3 | 1.7 | N | N | N |
| 13 | Non-invasive papillary UC, low grade | 1.7 | N | N | N | N | N | 1.2 | N | N | N | 2.4 | N | 7.5 | 1.5 | 1.2 | 0.1 |
| 14 | Urohelial Papilloma | 3.2 | N | 1.6 | N | 1.1 | N | 2.5 | 0.8 | 2.3 | 1 | 1.6 | 0.2 | 5 | N | 2 | N |
| 15 | Non-invasive papillary UC, low grade | N | N | N | N | 1.9 | N | 0.4 | N | 1.4 | 0.4 | N | N | 3 | 67.7 | N | N |
| 16 | Urothelial Papilloma | N | N | N | N | N | N | 0.9 | N | 2 | 2.2 | 1.5 | 2.7 | 1.6 | N | N | N |
| **17#** | **Non-invasive papillary UC, low grade** | **2.2** | **N** | **1.9** | **N** | **1.3** | **1** | **3** | **N** | **1.5** | **0.3** | **2.7** | **0.7** | **4.3** | **2.4** | **2.6** | **2** |
| **18#** | **Non-invasive papillary UC, low grade** | **1.8** | **4.8** | **0.1** | **0.7** | **1.2** | **0.6** | **1.5** | **0.6** | **1.6** | **1.3** | **2** | **3** | **1.9** | **2.6** | **1.5** | **1** |
| **19#** | **Non-invasive papillary UC, low grade** | **1.4** | **1** | **1.3** | **N** | **N** | **N** | **2** | **N** | **2.7** | **1** | **2.3** | **1.6** | **3.5** | **1.4** | **1** | **N** |
| 20 | Urothelial carcinoma in situ (CIS) | 4 | N | N | N | 5.8 | 0.4 | 0.6 | N | 2.4 | 1.8 | 1.7 | 4.3 | 2.4 | 1.9 | N | N |
| 21 | CIS with microinvasion | N | N | N | N | 0.5 | N | 6.5 | 0.7 | 0.8 | N | 2.2 | N | 9.7 | 9.4 | N | N |
| 22 | Non-invasive papillary UC, high grade | 1.5 | 0.2 | N | N | 0.3 | 3.3 | 2.5 | 9.4 | 16.4 | 1 | 4 | N | 13.3 | 0.2 | N | N |
| 23 | CIS | N | N | 1.5 | 0.4 | 0.7 | N | 1.2 | 0.6 | 1.2 | N | 1.7 | N | 1.9 | 0.6 | 1.5 | 1 |
| 24 | Invasive UC, high grade | 4.4 | N | 2 | 0.5 | 0.5 | N | 0.9 | N | 4 | 2.6 | 1.2 | 5.5 | 3.4 | 0.5 | 11 | N |
| 25 | Urothelial Papilloma | N | N | N | N | 1.3 | 0.5 | N | N | 1 | 2 | 0.5 | 0.5 | 0.5 | 1.1 | 0.5 | N |
| 26 | Invasive UC, high grade | 4.2 | N | N | N | N | N | 0.9 | N | 0.8 | 0.5 | 0.8 | 0.1 | 2.5 | 2.8 | N | N |
| 27 | Invasive UC, high grade | 1.1 | 0.3 | 1.1 | 0.1 | 2.2 | N | 3 | 0.2 | 8 | 4 | 2.2 | 0.3 | 4 | 1 | 2.5 | 1 |
| 28 | Invasive UC, high grade | 8.8 | N | 3.8 | N | 3 | N | 4 | N | 3 | 1.5 | 1.6 | 0.5 | 3 | 1.7 | 3.3 | 1 |
| 29 | Capillary Hemangioma | 1.9 | N | N | N | N | N | N | N | 0.7 | 1 | 0.6 | 3.2 | 2 | 2 | 1 | N |
| 30 | CIS | N | N | N | N | 1 | 0.4 | N | N | 2.5 | N | 2 | 0.3 | 4 | 1 | 2.7 | N |
| 31 | Non-invasive papillary UC, high grade | N | N | 0.1 | 5 | 0.6 | 1.7 | N | N | 0.5 | N | 0.2 | 0.2 | 6 | N | N | N |
| **32#** | **CIS** | **N** | **N** | **N** | **N** | **N** | **N** | **11** | **0.2** | **1** | **0.1** | **2** | **0.5** | **N** | **N** | **N** | **N** |
| **33#** | **Non-invasive papillary UC, low grade** | **0.1** | **0.1** | **0.2** | **N** | **4** | **0.3** | **N** | **N** | **2.6** | **N** | **1.7** | **0.4** | **1** | **0.2** | **4** | **0.6** |
| **34#** | **CIS** | **0.1** | **N** | **1.2** | **N** | **3** | **N** | **12.3** | **0.2** | **2.7** | **0.5** | **2.6** | **N** | **4.5** | **3.3** | **2.6** | **0.1** |
| 35 | Non-invasive papillary UC, high grade | N | N | N | N | 0.6 | N | N | N | 1 | 0.3 | N | N | 3.3 | 0.6 | N | N |
| 36 | Non-invasive papillary UC, high grade | 0.1 | 0.2 | N | N | 1 | N | N | N | 0.6 | 0.1 | 1 | N | 3.3 | N | 2.8 | N |
| 37 | Non-invasive papillary UC, high grade | N | N | 1.1 | N | N | N | 0.9 | N | N | N | 0.1 | 0.2 | N | N | N | N |
| 38 | CIS | N | N | 5 | N | N | N | N | N | N | N | N | N | N | N | N | N |
| 39 | Inverted urothelial papilloma | N | N | 2 | N | N | N | 3.7 | N | N | N | 0.2 | 0.2 | N | N | N | N |
| 40 | CIS with microinvasion | N | N | 7 | N | 0.3 | 0.1 | 2.7 | N | 2.8 | 0.2 | 0.7 | 0.1 | N | N | 4 | 0.1 |
| 41 | Urothelial Papilloma | N | N | 1.5 | 0.4 | 2.2 | N | 3.1 | N | N | N | 0.2 | 0.1 | N | N | N | N |
| 42 | CIS and Hemangiosarcoma | N | N | 3 | N | N | N | N | N | N | N | N | N | N | N | N | N |
| 43 | Non-invasive papillary UC, high grade | N | N | 2 | N | 0.1 | N | 4 | 0.1 | 3 | 0.5 | N | N | N | N | 2.7 | 0.2 |
| **44*** | **Non-invasive papillary UC, high grade** | **N** | **N** | **3** | **N** | **1.7** | **N** | **6** | **0.1** | **N** | **N** | **N** | **N** | **N** | **N** | **N** | **N** |
| 45 | Non-invasive papillary UC, high grade | N | N | N | N | 45 | N | N | N | N | N | 0.3 | N | N | N | 2.7 | N |
| 46 | Invasive UC, high grade | N | N | 3 | 0.7 | 3 | 0.1 | 5 | N | N | N | 0.3 | 2.3 | N | N | N | N |
| 47 | Invasive UC, high grade | N | N | 1.2 | N | N | N | N | N | 0.2 | 1.7 | N | N | N | N | 2 | N |
| 48 | Non-invasive papillary UC, low grade | N | N | 1.3 | 0.3 | 7.3 | N | N | N | 1 | 0.4 | 1 | 0.1 | N | N | 7 | N |
| 49 | CIS | 2 | N | 0.7 | N | 6 | N | 1.9 | N | 0.4 | N | 0.6 | N | 0.8 | N | 0.3 | N |
| 50 | Invasive UC, high grade | N | N | 2.1 | N | 3 | N | 9 | N | N | N | N | N | 0.3 | N | 0.2 | N |
| **51#** | **Non-invasive papillary UC, high grade** | **2** | **0.2** | **0.1** | **0.2** | **6** | **N** | **1.9** | **N** | **0.4** | **N** | **0.6** | **0.6** | **0.8** | **0.6** | **0.3** | **N** |
| 52 | CIS | N | N | 2.1 | 0.5 | 22 | N | 8 | 0.2 | N | N | N | N | 0.3 | N | 0.2 | N |

| Supplemental Table S1 provides a summary of the detected and quantified BPV and OaPV DNA and its transcripts in bladder tumor samples. N= negative; numbers represent copies/μL |
| --- |

The samples (1, 7, 8, 17, 18, 19, 32, 33, 34, 51) marked with the symbol **# (hash)** showed a mixed infection (positive for both BPV and OaPV); they have been utilized for WB of Figure 3, (1 to 10 respectively). The samples (9, 10, 11, 44) marked with the symbol *** (asterisk)** were positive to BPV only. These samples have been utilized for obtaining results of WB showed in Figure 3 too.
